# Injectability, Processability, Drug Loading, and Antibacterial Activity of Gentamicin-Impregnated Mesoporous Bioactive Glass Composite Calcium Phosphate Bone Cement In Vitro

**DOI:** 10.3390/biomimetics7030121

**Published:** 2022-08-28

**Authors:** Ming-Hsien Hu, Pei-Yi Chu, Ssu-Meng Huang, Bo-Sin Shih, Chia-Ling Ko, Jin-Jia Hu, Wen-Cheng Chen

**Affiliations:** 1 Bachelor Program for Design and Materials for Medical Equipment and Devices, Da-Yeh University, Changhua 515, Taiwan; 2 Orthopedic Department, Show Chwan Memorial Hospital, Changhua 500, Taiwan; 3 Department of Post-Baccalaureate Medicine, College of Medicine, National Chung Hsing University, Taichung 402, Taiwan; 4 Department of Pathology, Show Chwan Memorial Hospital, Changhua 500, Taiwan; 5 School of Medicine, College of Medicine, Fu Jen Catholic University, New Taipei City 242, Taiwan; 6 Department of Health Food, Chung Chou University of Science and Technology, Changhua 510, Taiwan; 7 National Institute of Cancer Research, National Health Research Institutes, Tainan 704, Taiwan; 8 Advanced Medical Devices and Composites Laboratory, Department of Fiber and Composite Materials, Feng Chia University, Taichung 402, Taiwan; 9 School of Dentistry, College of Dental Medicine, Kaohsiung Medical University, Kaohsiung 807, Taiwan; 10 Department of Mechanical Engineering, National Yang Ming Chiao Tung University, Hsinchu 300, Taiwan; 11 Department of Fragrance and Cosmetic Science, College of Pharmacy, Kaohsiung Medical University, Kaohsiung 807, Taiwan

**Keywords:** calcium phosphate bone cement (CPC), mesoporous bioactive glass, antibiotic, in vitro, biocompatibility

## Abstract

Calcium phosphate cement (CPC) is similar to bone in composition and has plasticity, while mesoporous bioactive glass (MBG) has the advantage of releasing Si, which can promote osteogenic properties and drug loading capacity. A sol–gel-prepared MBG micro-powder (mMBG) and further impregnated antibiotic gentamicin sulfate (Genta@mMBG: 2, 3, and 4 mg/mL) antibiotic were added to CPC at different weight ratios (5, 10, and 15 wt.%) to study CPC’s potential clinical applications. Different ratios of mMBG/CPC composite bone cement showed good injectability and disintegration resistance, but with increasing mMBG addition, the working/setting time and compressive strength decreased. The maximum additive amount was 10 wt.% mMBG due to the working time of ~5 min, the setting time of ~10 min, and the compressive strength of ~51 MPa, indicating that it was more suitable for clinical surgical applications than the other groups. The 2Genta@mMBG group loaded with 2 mg/mL gentamicin had good antibacterial activity, and the 10 wt.% 2Genta@mMBG/CPC composite bone cement still had good antibacterial activity but reduced the initial release of Genta. 2Genta@mMBG was found to have slight cytotoxicity, so 2Genta@mMBG was composited into CPC to improve the biocompatibility and to endow CPC with more advantages for clinical application.

## 1. Introduction

Complex bone defects caused by accident, infection, and aging are often accompanied by poor self-repair and must be restored with synthetic bone substitutes. An ideal synthetic bone substitute needs to be osteoconductive and osteoinductive at the repair site. Calcium phosphate bone cement, commonly known as CPC, is a mixture of tetracalcium phosphate (TTCP) and dicalcium phosphate anhydrous (DCPA) powder with a Ca/P atomic ratio of about 1.67. It reacts with a hardening solution of dilute phosphate to form a slurry to allow solidification in situ. The reaction product of CPC bone cement is mainly hydroxyapatite (HA), which is ideal as a biomimetic cortical bone tissue [1]. The slurry-like bone filler CPC is injectable and plastic before the operative working time [2], and can be used more effectively in irregular-shaped bone defects. It is among the most widely used bone repair materials in clinical practice, but there are still problems, such as insufficient mechanical strength, a long setting time, and easy disintegration when in contact with blood [3]. Many strategies can be used to overcome these problems, such as powder vacuum sintering, surface pretreatment to speed up the reaction, and the addition of polymeric binders [4]. To meet more clinical needs by modifying CPC or by applying additional functions, recent studies have added organic or inorganic filler composites with different functions to the CPC to further improve the compressive strength, or to promote the degradation of CPC [5]. For example, the addition of hydrogel microspheres causes the degradation of hydrogel, thereby increasing the porosity of the CPC, which induces substantial bone growth after a period of implantation [6]. The CPC composites containing biodegradable carriers promote the ingrowth of blood vessels and tissues, and are functionalized by carrying antibiotics, anti-inflammatory drugs, and bone-promoting factors in the carriers. Therefore, a series of CPC composite bone cements have been developed to contribute to the widespread use of CPC [7,8].

Mesoporous bioactive glasses (MBGs) are promising materials for regenerative medicine, due to their favorable properties, including biocompatibility, osteoinduction, and degradability [9,10,11]. These key properties, along with their surface area, pore structure, and pore volume, are highly recommended for tissue regeneration [9,10,11,12,13]. However, systematic studies on the performance of MBG/CPC composite bone cement covering a wide range of possible drug-loading compositions are lacking [12,13,14,15,16]. The key features of MBGs are a highly ordered channel-like pore systems in the 5–20 nm range, a large pore volume, and significantly increased specific surface area to facilitate cell adhesion, proliferation, and drug impregnation; they also show enhanced biodegradation [10,11,12,13]. By controlling MBG degradation, ion and drug release can be controlled to modulate tissue regeneration. MBGs can utilize physical or chemical bonding to adsorb drug molecules onto surfaces or pores, and release them [13]. Irregularly shaped MBG micro-powders (mMBGs) with a size of approximately 100–500 µm were synthesized using a sol–gel method in an acidic environment [14]. MBGs have certain limitations in repairing bone damage in vivo, especially for load-bearing bone tissue damage, because it cannot form an accurate shape to repair the damage, and the powder of MBG has no strength [15]. Therefore, MBG as a drug carrier can be composited into CPC to form an injectable plastic MBG/CPC bone cement, which can repair irregular bone defects and provide additional functions [16].

Although the combined use of CPC and drug-impregnated mMBG can control drug release, improve biocompatibility, promote bone growth, and address the limitations of their respective applications, it also affects the physicochemistry, microstructure, injectability, hardening properties, and mechanical strength [17,18]. Although MBG/CPC bone cements have been studied before, the exact composition can vary due to the different properties of CPC bone cements, and the drug loading can be considered as different [16,17,18,19]. Therefore, the purpose of this study was to prepare different ratios of gentamicin-impregnated mMBG and composite them into CPCs to develop an mMBG/CPC bone cement. The study also aimed to study the physicochemical properties, mechanical strengths, antibacterial activities, and biocompatibilities of the new mMBG/CPC bone cement.

## 2. Materials and Methods

### 2.1. Raw Materials

The raw materials used in this study include tetracalcium phosphate (Ca_4_P_2_O_9_, TTCP; Realbone Technology Co., Ltd., Kaohsiung, Taiwan), surface-modified dicalcium phosphate anhydrous (sm-DCPA; Realbone Technology Co., Ltd., Kaohsiung, Taiwan), calcium nitrate (Ca(NO_3_)_2_ 4H_2_O, Katayama Chemical Industries Co., Ltd., Osaka, Japan), nitric acid (HNO₃, PANREAC, Barcelona, Spain), tetraethyl orthosilicate (Si(OC_2_H_5_)_4_, TEOS, ACROS ORGANICS, Belgium), triethyl phosphate ((C_2_H_5_)_3_PO_4_, TEP, Alfa Aesar, Johnson Matthey Company, Devens, MA, USA), Pluronic^®^ F-127 (H(C_2_H_4_O)_x_(C_3_H_6_O)_y_(C_2_H_4_O)_z_OH, Sigma-Aldrich, St. Louis, MO, USA), ethanol (CH_3_CH_2_OH, J. T. Baker, Radnor, PA, USA), and antibiotic gentamycin (Genta; Siu Guan Chemical Industrial Co., Ltd., Chiayi, Taiwan).

### 2.2. Preparations of CPC, mMBG, Genta@mMBG, and mMBG/CPC Composite Bone Cements

#### 2.2.1. CPC Preparation

The pulverization process of TTCP and the surface modification of sm-DCPA have been shown in our previous study [4]. The process was as follows: 16.6 g of TTCP, 12.4 g of sm-DCPA, and 117 g of alumina spheres were added to a PE bottle and mixed for 24 h to prepare the CPC.

#### 2.2.2. MBG Micro-Powder and Antibiotic-Impregnated mMBG (Genta@mMBG)

In this experiment, mMBG was prepared using an acid-catalyzed method based on the sol–gel method. The process was as follows: 1 g of surfactant F-127 and an acid catalyst of 2 M nitric acid (1 g) were mixed with the precursor material of SiO_2_-CaO-P_2_O_5_ (the molar ratio of Si:Ca:P = 80:15:5), which was prepared by dissolving 6.7 g of TEOS, 1.43 g of calcium nitrate, and 0.73 g of TEP in 60 g of ethanol and stirring for 24 h. The above sol–gel was impregnated with a polyurethane sponge, dried at 100 °C, calcined to 600 °C at a rate of 10 °C/min, and held for 2 h. The sintered cake was pulverized and filtered through a 325 mesh sieve to obtain an irregularly shaped mMBG powder. Gentamicin at a stock concentration of 40 mg/mL was mixed into double distilled water (ddH_2_O) to adjust 50 mL of different concentrations of gentamicin (2, 3, and 4 mg/mL) in water; gentamicin was then mixed with mMBG to make a liquid ratio mixture of 1/50 (g/mL). After stirring for 24 h, the antibiotic-impregnated powder was filtered and dried at room temperature for 24 h to obtain drug loading Genta@mMBG (2Genta@mMBG, 3Genta@mMBG, and 4Genta@mMBG).

##### 2.2.3. mMBG/CPC and 2Genta@mMBG/CPC Composite Bone Cement

CPC powder was mixed with different weight ratios (5, 10, and 15 wt.%) of mMBG and 10 wt.% of Genta@mMBG mixed with 0.67 M phosphate solution hardener. The pH of the hardener was adjusted to 6.02. An mMBG/CPC slurry was formed at a powder-to-liquid ratio of 0.8 g/380 μL. This was mixed well within 1 min, and the slurry was poured into a mold and left to set for 3 min. During sample preparation, the sample was kept under a pressure of 0.7 MPa (100 psi), and pressed into a cylindrical sample with a height of 12 mm and a diameter of 6 mm. It was then demolded, and the following physical and chemical properties were compared.

### 2.3. Characterization of MBG and Antibiotic Impregnated Genta@mMBG

The powder morphology of the mMBG particles was observed via transmission electron microscopy (JEM-2100F, JEOL, Tokyo, Japan) operated at 200 kV to characterize the internal structures and micrographs of mMBG and Genta@mMBG particles. The specific surface area and changes between mMBG and Genta@mMBG particles were determined using the Brunauer–Emmett–Teller (BET) method. The nitrogen adsorption and desorption isotherm data were obtained at −196 °C on a constant volume adsorption device (ASAP2020, Micromeritics, Norcross, GA, USA).

### 2.4. Characterization of mMBG/CPC and Genta@mMBG/CPC Composite Bone Cements

#### 2.4.1. X-ray Diffraction (XRD) and Fourier Transform Infrared Spectroscopy (FTIR) Analysis

XRD was used to analyze the crystallinity and phases of samples. XRD was used to analyze whether the crystallization of mMBG or Genta@mMBG on CPC was affected. The bone cement after the reaction was ground into powder and analyzed via XRD diffractometer (XRD-6000, Shimadzu, Kyoto, Japan). Diffraction conditions using Ni-filtered Cu target Kα were typically operative at 30 kV, 20 mA, and scanning angles were in the range of 20–60° with a scanning 2θ rate of 2°/min. The relative XRD patterns were determined by comparing the different diffraction peaks with the JCPDS standard diffraction in the database file.

FTIR spectra were used to analyze the functional group changes of CPC after composite mMBG and Genta@mMBG. The sample was made by mixing the powder with KBr at a ratio of 1/100 (g/g) and pressing it into a translucent circular sheet with a diameter of approximately 12 mm. IR spectra were obtained by detecting changes of translucent sheets in transmittance (or absorption) intensity as a function of frequency in an FTIR spectrometer (Nicolet iS5, Thermo Fisher Scientific, Waltham, MA, USA).

#### 2.4.2. Working/Setting Time, Injectability, and Dispersibility

When the bone cement was mixed and the slurry was gradually hardened, no mutual adhesion was found between the powders in the slurry; i.e., the two slurries could not be aggregated into a block, which was an indicator that could be used to measure the working time. The setting time was measured according to a dental phosphoric acid standard, ISO 9917-1, by pressing down the sample vertically with a 400 g Gillmore needle at 37 °C and 60–70% humidity until no visible indentation was found on the surface; i.e., the hardening time was recorded.

The test of injectability and disintegration was performed by putting the bone cement after mixing for 1 min into a 5 mL syringe within 3 min, and immediately applying 250 N vertically to the syringe barrel to inject the bone cement into 37 °C ddH_2_O. The change of bone cement after injection into the water was observed by taking pictures. A successful injection meant that it can be injected. If no disintegration phenomenon was observed after 15 min of immersion, then it has disintegration resistance.

#### 2.4.3. Compressive Strength and Fracture Surface Observation

The sample mold used in this study had a diameter of 6 mm and a height of 12 mm, and was tested according to ASTM F451-16. The sample was immersed in Tris-buffer solution artificial body fluid at a ratio of 1 g/10 mL. It was placed at 37 °C for 1 day and then taken out. The compressive strength of the wet specimens was measured using a universal testing machine (HT-2402, Hung Ta, Taichung, Taiwan) at a crosshead speed of 1 mm/min.

The morphology of composite bone cement after compression was observed using a scanning electron microscope (SEM; S-3000N, HITACHI, Tokyo, Japan) equipped with an energy dispersive spectrometer (EDS) to observe the distribution of Si, and to analyze whether the MBG was well compounded into the CPC.

### 2.5. Antibacterial Abilities

*Staphylococcus aureus* (*S. aureus*; ATCC No. 25923) and *Escherichia coli* (*E. coli*; ATCC No. 10798) were cultured in tryptic soy broth (TBS). The bacterial suspension was diluted to achieve an optical density of 0.2 (equivalent to ~1.0 × 10^7^ cells/mL on average) at 595 nm (OD_595_). This value was confirmed with an enzyme-linked immunosorbent assay (ELISA) reader (EZ Read-400, Biochrom, Holliston, MA, USA). The bacterial suspensions of *S. aureus* and *E. coli* were subsequently diluted to achieve an OD_595_ value of 0.2. First, the bacterial liquid cultured with TBS was evenly coated on the surface of the agar, and Genta@mMBG and Genta@mMBG/CPC were used to form cylindrical samples with a diameter of 6 mm and a height of 3 mm. The samples were then attached to the plate and incubated at 37 °C for 24 h. The zone of inhibition was observed and measured. In terms of antibacterial quantification, 0.2 g of the sample was mixed with 2 mL of bacterial suspension and cultured at 37 °C for 1, 2, 3, and 4 days. Approximately 100 μL of bacterial suspension was removed at each time point, and an equal amount of TBS was added to the original shaker tube. The absorbance of OD_595_ was measured, and the antibacterial effect of the samples was quantified.

### 2.6. In Vitro Cytotoxicity Tests

The L929 cell line of neonatal mouse fibroblasts was provided by the National Institutes of Health, Miaoli, Taiwan, and used for cytotoxicity assays. The testing procedure was performed according to ISO 10993-5:2009. L929 cells were cultured with culture medium in an incubator at 37 °C and 5% CO_2_, and subcultured when cell concentrations were between 0.8 × 10^6^ and 1.0 × 10^6^ cells/mL. The medium used was Minimal Essential Medium alpha medium (Gibco, Thermo Fisher Scientific Inc., Waltham, MA, USA) containing 10% horse serum, changed every 2 days of culture.

The samples prepared for cell culture were sterilized via autoclaving at 121 °C and 1.05 kg/cm^2^ (15–20 psi; TOMIN, TM-328, Taipei, Taiwan). Sterilized mMBG/CPC composite, high-density polyethylene (HDPE) negative control, and 15 vol.% dimethyl sulfoxide (DMSO; Sigma-Aldrich, St. Louis, MO, USA) were added into the medium. The extraction ratio of sample to medium was set to 1 g/5 mL, and the samples were placed in a 37 °C incubator for 24 h to extract the medium for cytotoxicity detection.

For quantitative cytotoxicity arrays, 100 μL of suspension containing L929 1 × 10^4^ cells was transferred to a 96-well microplate and incubated at 37 °C for 24 h. Media was removed and 100 µL of sample extract was added, then L929 cells were incubated in a 37 °C incubator for 24 h and mixed with 50 µL of XTT Cell Proliferation Assay Kit (Biological Industries, Kibbutz Beit Haemek, Israel) for a 4 h extension reaction. Afterwards, they were processed with another ELISA reader (SPECTROstar Nano, BMG LABTECH, Offenburg, Germany). The measured OD_490_ absorbance was proportional to the cell viability.

In the qualitative cytotoxicity test, the control group extract and medium were prepared in the same way as in the quantitative test. Approximately 1000 μL of cell suspension were collected, and L929 cells were seeded into a 48-well microplate at a cell concentration of 1 × 10^5^ cells/well. The original medium was cultured in an incubator at 37 °C and 5% CO_2_ for 1 day. Then, the medium was removed. Sample extract (100 μL) was added and cultured for 24 h, and cell morphology was observed under an inverted microscope (IVM-3AFL, SAGE VISION Co., LTD, New Taipei City, Taiwan).

### 2.7. Statistical Analysis

Analysis of variance (ANOVA) was performed using IBM SPSS Statistics (version 20, IBM Corp., Armonk, NY, USA) for the measurement of working/setting time, compressive strength, and antibacterial abilities. ANOVA was used to determine whether the differences among the means of multiple groups were significant. The estimates of two different variables were used to compare the differences.

## 3. Results and Discussion

### 3.1. Characterization of Genta@mMBG/CPC Composite

#### 3.1.1. Injectability, Dispersibility, and Working/Setting Time of Genta@mMBG/CPC Composite

**Figure 1:**
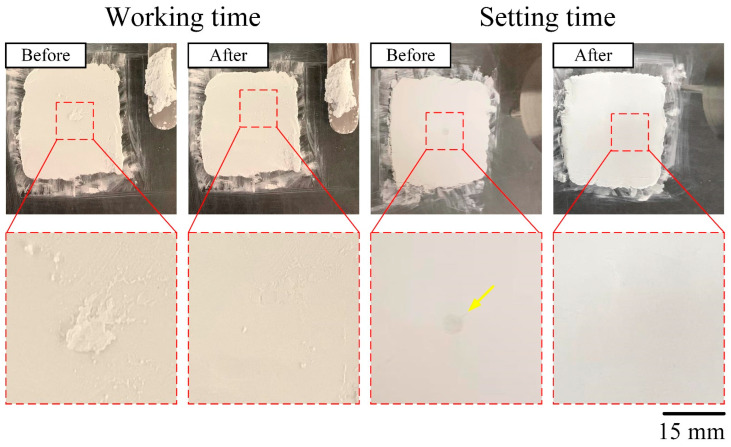
Comparison of optical images of test samples before and after Gillmore needle acupuncture; penetration testing was performed to measure the difference between the working and setting times of the mMBG/CPC composite bone cement. (Arrow shows the indentation of the Gillmore needle before setting).

The working time is indicated when the slurry gradually hardens and there is no further mutual adhesion between the slurries. The initial strength was tested with a Gillmore needle, no obvious indentation was found on the surface, and the setting time was recorded. Optical image comparison of the mMBG/CPC composite bone cement to measure the difference between the working and setting times is shown in Figure 1.

**Figure 2:**
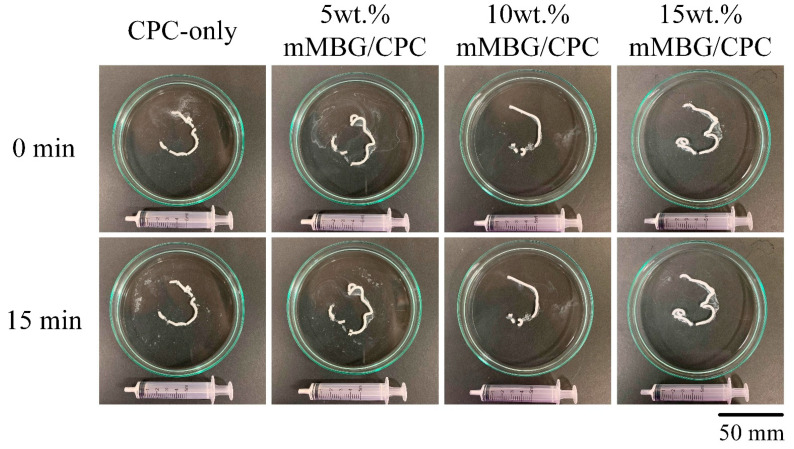
Analysis of slurry injectability (**upper row**) and dispersibility (**lower row**) of CPC-only and mMBG/CPC composite bone cements with different mMBG ratios of 5, 10, and 15 wt.%.

Considering that CPC bone cement has the advantage of being able to pipe the slurry before the CPC sets, it can be applied as a minimally operative procedure, and it would perfectly fit the repair site. Its anti-washout and anti-dispersion properties in solution are critical for clinical applications. The addition of fillers to the CPC may alter the properties of the CPC. They should be measured because they affect the handling properties or the acceptable range of cement work and setting time. The anti-extrusion force may hinder the application of CPC for the injection process. In this study, the required injection time, including mixing and loading times, was controlled within 3 min, and the injection results are shown in Figure 2. All groups could be injected smoothly without an obvious blockage of CPC residues, and no obvious powder dispersion phenomenon was observed after the CPC was injected into ddH_2_O. At the same time, the mMBG/CPC composite showed no obvious disintegration phenomenon after soaking for 15 min, indicating that the ratio of mMBG in CPC did not significantly hinder the original injection or anti-disintegration ability of CPC.

#### 3.1.2. Working/Setting Time, Compressive Strength, and Fracture Surface Observation of GentaM/CPC Composite

The working time, setting time, and compressive strength test results of CPC-only and mMBG/CPC composite bone cements are shown in Table 1. The mMBG addition shortened the working and setting times (*p* < 0.05), and the time decreased with the increasing content of mMBG added (*p* < 0.05). The working and setting times of the CPC composite bone cement range for each group were 3–9 and 8–13 min, respectively. The working time of injectable bone cement is the duration for which the cement remains at a more or less constant and ideal viscosity for delivery to in situ restoration and penetration of the restoration. Based on the phase change of the CPC solidification reaction, the setting time provides an initial strength for overcoming anti-rushing and dispersion before the strength reaches its peak. According to the expectations of consulting clinicians and literature [8,19,20,21], the ideal working time of CPC composite bone cement needs to be controlled in the range of 4–10 min, and the setting time needs to be in the range of 10–20 min. The developed 10 wt.% mMBG/CPC composite with the highest mMBG content achieved the expected runtime.

**Figure 3:**
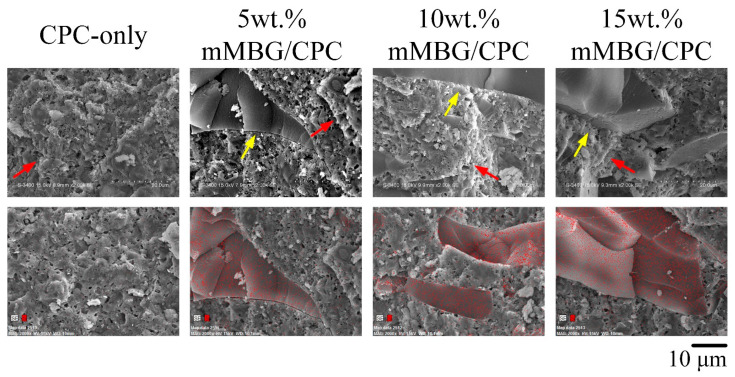
Fractured surfaces of the specimen after compression; SEM images and Si elemental mapping (red arrow: HA, yellow arrow: mMBG) of control CPC-only and mMBG/CPC composite bone cements with different mMBG ratios immersed in Tris-buffer for 1 day.

Considering that the value of the compressive strength of the human trabecular bone is 0.2–16 MPa [22,23,24], the ideal strength of the implant material needs to be at least close to that of trabecular bone, to judge whether the implant material meets the needs of clinical applications. The compressive strength of the mMBG/CPC composite bone cement after immersion in Tris-buffer solution for 1 day was between 42 and 75 MPa, indicating that each group was suitable for clinical application. Figure 3 shows the fracture images and Si elemental mapping of mMBG/CPC composites with different mMBG ratios after soaking in Tris-buffer for 1 day. A coral reef-like crystal structure formed on the surface of each group; this was the product of apatite after the CPC reaction. Smooth lumps were observed in the mMBG-added CPC composite, and further Si mapping ensured that the main component was silicon. Considering that the distribution of mMBG among the groups was random rather than uniform, a further decrease in compressive strength was observed. According to the comprehensive consideration of working/setting time and compressive strength, the optimal addition amount of mMBG in this study was 10 wt.%. Therefore, subsequent analyses used this addition to impregnate Genta in mMBG (Genta@mMBG), which was then composited into CPC.

### 3.2. Characterization of mMBG and Genta-Impregnated mMBG

#### 3.2.1. Antibacterial Abilities of mMBG and Genta@mMBG

**Figure 4:**
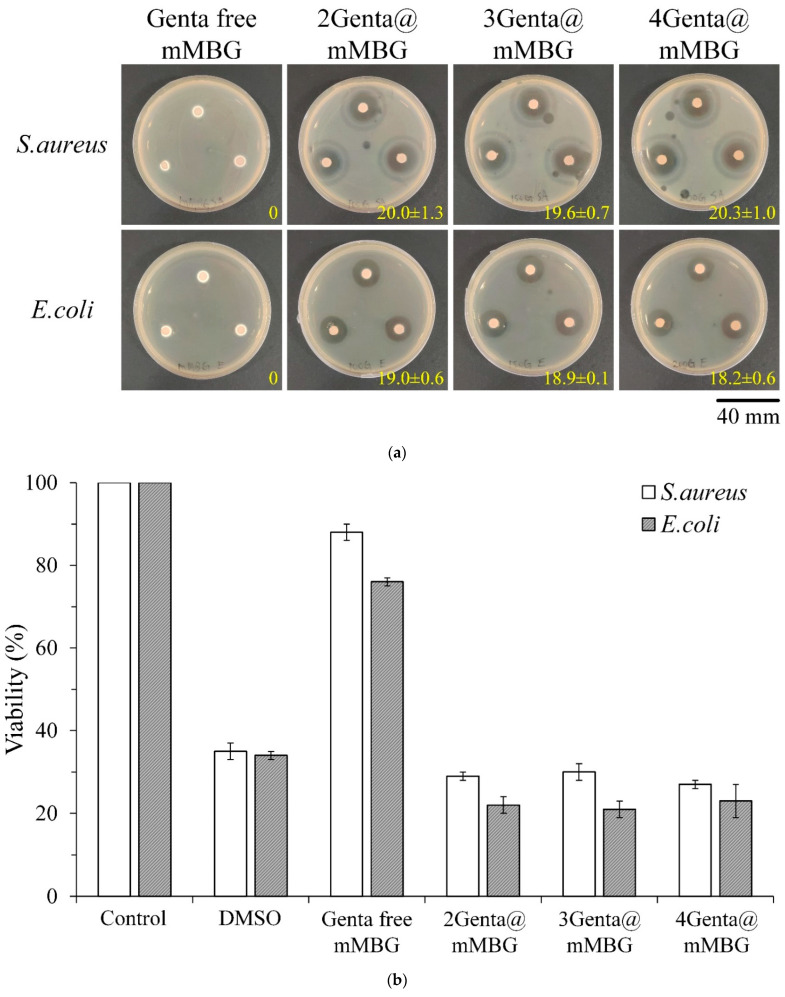
(**a**) Zone of inhibition and (**b**) relative quantitative antibacterial activity against Gram-positive *S. aureus* and Gram-negative *E. coli* in the presence of control Genta-free mMBG and mMBG impregnated with Genta at different concentrations for 1 day (*n* = 3).

Figure 4 shows the qualitative and quantitative antibacterial activities of the different concentrations of Genta-impregnated mMBG against *S. aureus* and *E. coli.* Results of the inhibition zone test in Figure 4a showed the obvious inhibition ability of each group on microbial growth, and the measured inhibition zone was similar. Results of the quantitative test in Figure 4b showed that the bacterial survival rates of each group were still similar, indicating that the drug concentration released by the 2Genta@mMBG group reached a good bacteriostatic ability that was similar to that of the positive control DMSO. Even if the concentration of Genta was increased to 4Genta@mMBG, the best bacteriostatic effect was obtained with 2Genta@mMBG. Therefore, the antibiotic concentration group, 2Genta@mMBG, with the least burden of a further application for clinical application, was selected for subsequent composite CPC experiments.

#### 3.2.2. SEM, TEM, and BET of MBG and Genta-Impregnated mMBG

**Figure 5:**
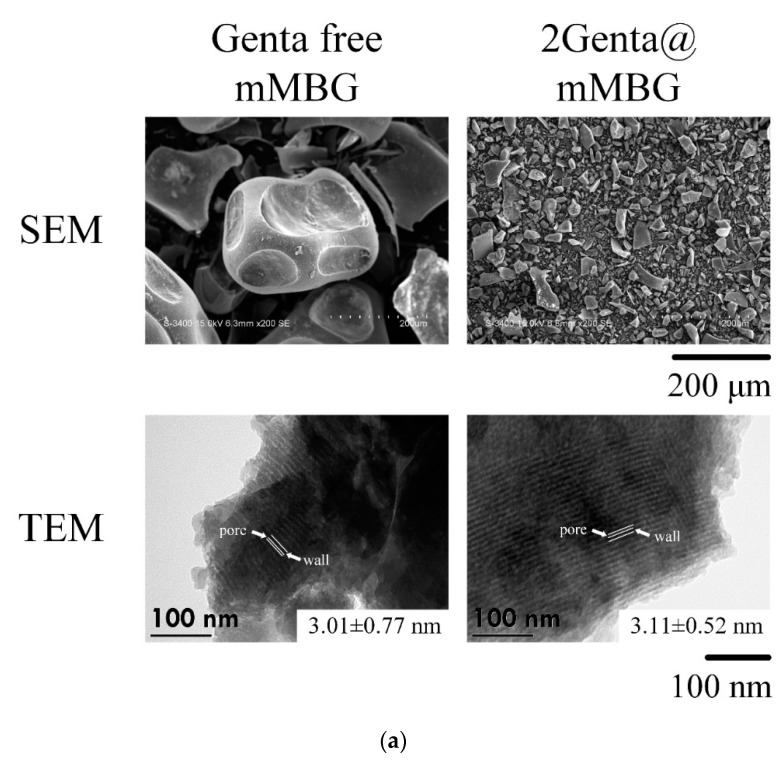

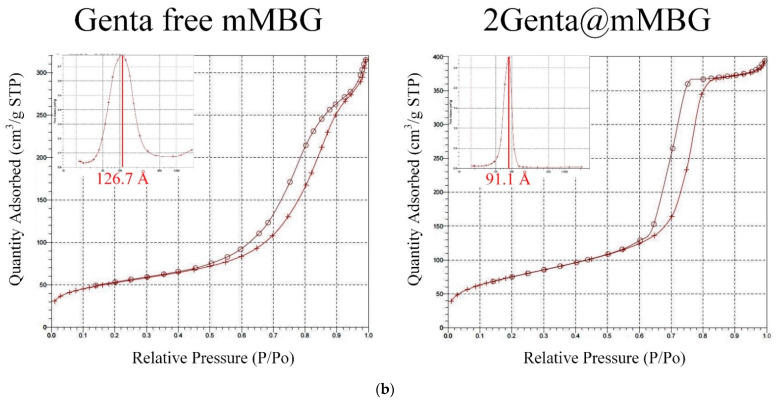
(**a**) SEM, TEM micrographs, and (**b**) nitrogen adsorption (+)/desorption (o) curves and pore size/pore volume distribution analysis diagram of Genta-free mMBG and 2Genta@mMBG.

Figure 5a shows the microstructural images of mMBG before and after impregnation with Genta. The SEM analysis shows that the mMBG particles before drug loading are large and irregular in shape, and with a particle size of approximately 100–500 µm. Several large concave pores were obvious on the surface, and some particles showed interconnected pore structures, which were related to the inheritance of polyurethane sponge as a dipping template for sintering. After drug loading, the particles became significantly smaller, the average particle size was less than 100 μm, and the shape was broken and irregular. As mMBG was soaked and agitated for a day, particle collisions occurred, resulting in the refinement of the mMBG particles. Images from TEM showed that even Genta-impregnated mMBG had mesopores neatly arranged in a grid. Considering that Genta impregnation was not observed via SEM and TEM, the subsequent nitrogen adsorption/desorption was used for drug loading analysis.

Figure 5b shows the nitrogen adsorption/desorption curves and pore size/pore volume distribution of mMBG before and after impregnation with Genta. All the curves of the groups are type IV isotherm curves, indicating that the two mMBGs have mesoporous structures before and after impregnation with the drug [25]. The type of hysteresis loop of mMBG before and after impregnation with Genta was the H1 type, indicating that the pore type was a neatly arranged cylindrical channel [26]. The pore size distribution map calculated using BJH theory was affected by the concave pores of different sizes on the surface. Thus, a pore size distribution with an average pore diameter of 126.7 Å (12.67 nm) before drug impregnation was wider.

Table 2 shows the summary results of specific surface area, pore volume, and pore size obtained via BET testing. After impregnation of mMBG with Genta, the specific surface area and pore volume increased, but the mesopore size decreased. The particle refinement after mMBG impregnation increased the specific surface area, especially the mMBG surface, and the dispersed Genta was also exposed in the internal pores of mMBG, thereby increasing the total pore volume and reducing the average pore size.

### 3.3. Characterization of mMBG/CPC and Genta@mMBG/CPC Composites

#### 3.3.1. FTIR and XRD of mMBG/CPC and Genta@mMBG/CPC Composites

**Figure 6:**
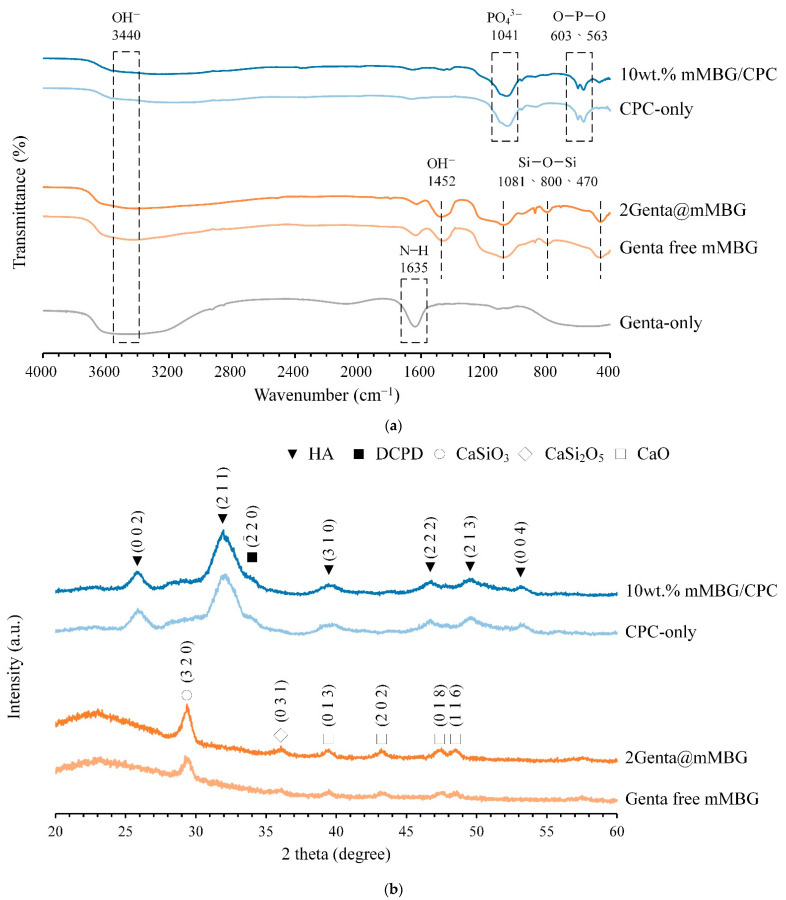
(**a**) FTIR and (**b**) XRD analysis of mMBG before (Genta-free) and after impregnation with Genta (2Genta@mMBG), and the CPC-only and 10 wt.% mMBG/CPC composite bone cement after 24 h immersed reaction.

The infrared spectra of mMBG before and after the impregnation of Genta and further composite CPC bone cement are shown in Figure 6a. Hydroxyl groups (OH^−^) can be found in mMBG at 1452, 1635, and 3440 cm^−1^ [27], indicating that the prepared MBG was hydrophilic [28]. At 470, 800, and 1081 cm^−1^, the non-stretching, symmetric, and asymmetric stretching vibration peaks of silicate (Si-O-Si) response are shown, respectively; these are the main absorption bands of MBG [29,30]. The N-H functional group in Genta should be displayed at 1635 cm^−1^ [31], but this cannot be verified in the 2GentaM group, because the N-H absorption band of Genta overlapped with broad Si-O-Si bands of mMBG. Then, after soaking the mMBG/CPC composite of 10 wt.% mMBG/CPC in Tris-buffer for 1 day, the O-P-O (ν_4_ mode in PO_4_^3–^) bending vibrations of HA can be seen at 563 and 603 cm^−1^. The asymmetric bending vibration of HA was found at 1041 cm^−1^ (ν_3_ mode in PO_4_^3−^) [32,33,34]. These bands indicated that the addition of mMBG does not affect the changes in CPC functional group vibrations. Figure 6b shows the XRD analysis of 10 wt.% Genta-free mMBG, 2Genta@mMBG, and mMBG further composite CPC. A broad SiO_2_ diffraction peak was observed between 20° and 30° (2*θ*) before and after mMBG impregnation with Genta, thereby indicating that mMBG is an amorphous structure [35,36]. After comparison with the database, mMBG observed the diffraction plane of CaSiO_3_ at 29.42° (JCPD 45-0156) [37,38]. Then, after soaking 10 wt.% mMBG/CPC in Tris-buffer for 1 day, the main diffraction planes of HA, such as (002), (211), (310), and (222), can be observed (JCPD 09-0432) [39], indicating that the addition of mMBG did not affect the phase transition of the CPC reaction into the HA phase.

#### 3.3.2. Antibacterial Abilities of Genta@mMBG/CPC Composite

**Figure 7:**
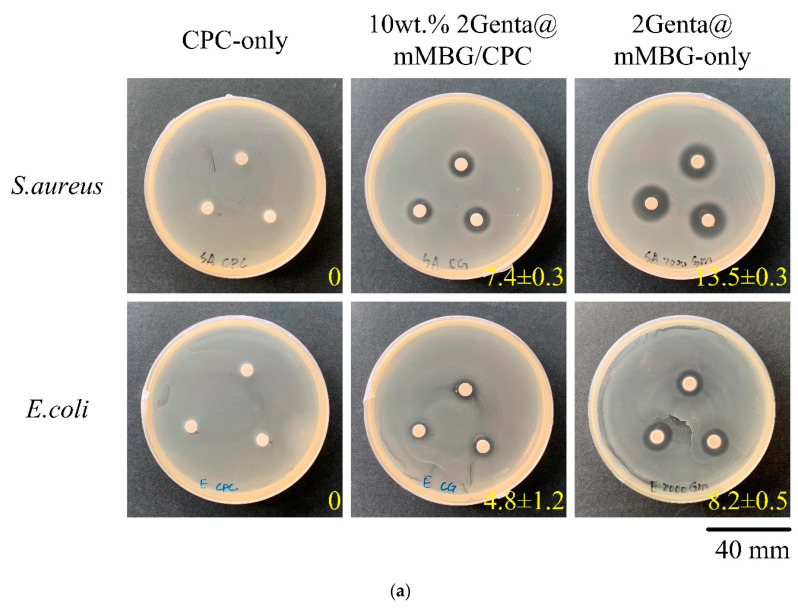

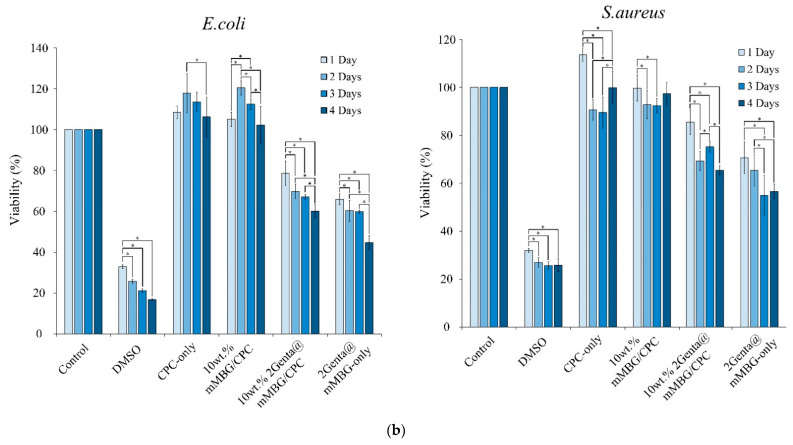
Antibacterial activities of Genta-loaded mMBG (2Genta@mMBG), CPC-only, 10 wt.% mMBG/CPC, and 10wt.% 2Genta@mMBG/CPC composite bone cements against *S. aureus* and *E. coli* for (**a**) a zone of inhibition of 1 day and (**b**) quantitative analysis for 1–4 days (*n* = 3; * indicates significantly different *p* < 0.05).

Figure 7a is the qualitative antibacterial test of 2Genta@mMBG and 10 wt.% 2Genta@mMBG/CPC composite. The inhibition zone after the 2Grenta@mMBG composite CPC was smaller than before compositing, indicating that combining Genta-impregnated mMBG into CPC reduced the initial antibiotic release of Genta. In addition, the antibacterial quantitative test for 1–4 days is shown in Figure 7b. The antibacterial effect of composite 10 wt.% 2Genta@mMBG/CPC was worse than that of 2Genta@mMBG-only, although it still had good antibacterial activity.

#### 3.3.3. Cytotoxicity of mMBG and Antibiotic-Impregnated 2Genta@mMBG and mMBG/CPC Composites

**Figure 8:**
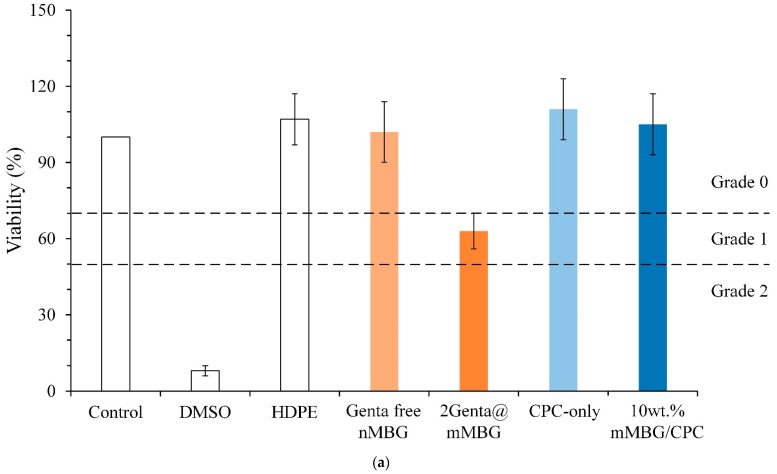

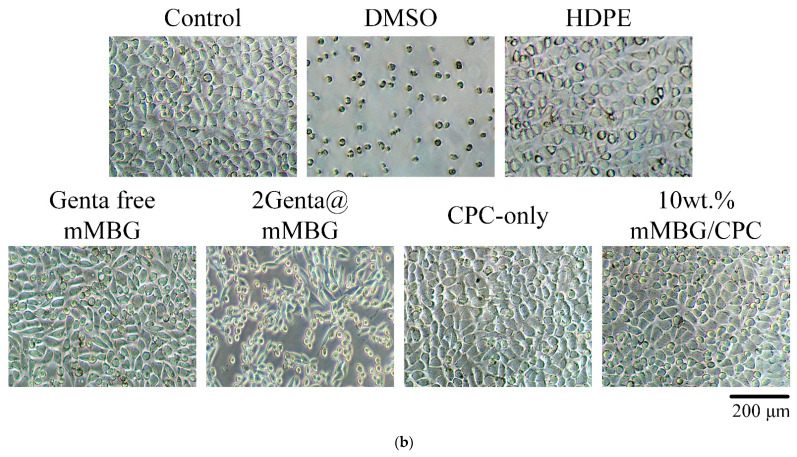
Extracts cultured with L929 cells for 1 day; (**a**) quantitative (*n* = 3) and (**b**) qualitative analysis of Genta-free mMBG and antibiotic impregnation (2Genta@mMBG); CPC-only, and 10 wt.% mMBG/CPC composite bone cement.

Figure 8 shows the quantitative and qualitative analyses of cytotoxicity before and after mMBG was loaded with Genta, and the composite CPC extract was cultured with L929 cells for 1 day. Cell viability between 50% and 70% indicated slight cytotoxicity (Grade 1) according to ISO 10993-5 [40]. If the biomaterial extract showed no evidence of causing cell lysis or toxicity, and if the cell cultures exposed to the test item did not show greater than mild reactivity (Grade 2), then the biomaterial can be considered to be cytocompatible. Considering that 2Genta@mMBG belongs to Grade 1 cytotoxicity rather than other groups of Grade 0 (no reactivity), it must be added to other materials to improve biocompatibility if it is used in clinical restoration. Figure 8b shows the qualitative analysis of cytotoxicity in each group. The results of all the groups are the same as those of the quantitative analysis of cytotoxicity.

## 4. Conclusions

Antibiotic-impregnated bioactive glass composite calcium phosphate bone cement was investigated. The addition of 5, 10, and 15 wt.% mMBG of mMBG/CPC composite bone cement did not affect the injectability of bone cement, and it still exhibited good anti-dispersion properties. However, considering that the working setting time and the compressive strength of the mMBG/CPC composites decreased with the increase of mMBG concentration, 10 wt.% mMBG was chosen to be added to mMBG/CPC. The working time was about 5 min, and the setting time was about 10 min. The FTIR and XRD results showed that the addition of mMBG did not hinder the formation of apatite. In addition, from the bacteriostatic test, in the mMBG impregnated with 2 mg/mL gentamicin, the minimum amount of Genta added in the 2Genta@mMBG group had good bacteriostatic activity, but less cytotoxicity. After 2Genta@mMBG composite CPC bone cement, 10 wt.% 2Genta@mMBG/CPC composite bone cement was observed to reduce the initial antibiotic burst release of Genta. Although 10 wt.% 2Genta@mMBG/CPC composite bone cement was not as effective as 2Genta@mMBG alone, it still had good antibacterial properties. Overall, 10 wt.% 2Genta@mMBG/CPC composite bone cement is antibacterial and does not significantly delay the CPC response, making it a good candidate for bone replacement materials.

## Figures and Tables

**Table 1:** Measured working and setting times, and compressive strength of CPC composite bone cements with different mMBG ratios. (*n* = 10).

Samples	Working Time (min)	Setting Time (min)	Compressive Strength (MPa)
CPC-only	9.81 ± 0.33	13.42 ± 0.66	75.40 ± 9.12
5 wt.% mMBG/CPC composite bone cement	7.62 ± 0.19 *	11.37 ± 0.65 *	70.32 ± 9.57
10 wt.% mMBG/CPC composite bone cement	5.24 ± 0.47 *	9.59 ± 0.42 *	50.97 ± 8.36 *
15 wt.% mMBG/CPC composite bone cement	3.12 ± 0.20 *	8.44 ± 0.31 *	42.37 ± 7.46 *

*, *p* < 0.05 indicates that the group is significantly different from the control CPC-only group.

**Table 2:** Analysis of specific surface area, pore volume, and pore size of Genta-free mMBG and 2Genta@mMBG.

Samples	Surface Area (m^2^/g)	Pore Volume (cm^3^/g)	Pore Size (nm)
Genta-free mMBG	187.60	0.45	9.54
2Genta@mMBG	274.45	0.59	8.57

## Data Availability

Data is contained within the article.
